# The feasibility of implementing the ICHOM Standard Set for Hip and Knee Osteoarthritis: a mixed-methods evaluation in public and private hospital settings

**DOI:** 10.1186/s41687-018-0062-5

**Published:** 2018-08-01

**Authors:** Ilana N. Ackerman, Bernarda Cavka, Jacob Lippa, Andrew Bucknill

**Affiliations:** 1 Department of Epidemiology and Preventive Medicine, Monash University, Melbourne, Australia; 2 Department of Medicine (Royal Melbourne Hospital), The University of Melbourne, Melbourne, Australia; 3 Physiotherapy Department, The Royal Melbourne Hospital, Melbourne, Australia; 4 International Consortium for Health Outcomes Measurement, Boston, USA; 5 Providence St Joseph Health, Seattle, USA; 6 Department of Orthopaedic Surgery, The Royal Melbourne Hospital, Melbourne, Australia

**Keywords:** Osteoarthritis, Outcomes assessment, Patient reported outcomes, Total knee replacement, Total hip replacement

## Abstract

**Background:**

There is growing international momentum for standardising patient outcome assessment and using patient-reported outcome measures (PROMs) to capture outcomes that matter to patients. The International Consortium for Health Outcomes Measurement (ICHOM) Standard Sets were developed to capture the outcomes of care for costly conditions including osteoarthritis. This study evaluated the feasibility of implementing the ICHOM Standard Set for Hip and Knee Osteoarthritis in ‘real world’ public and private hospital settings.

**Methods:**

A mixed-methods design was used to capture comprehensive data on patient outcomes, implementation costs, and the implementation experiences of patients, clinicians and administrative staff. The ICHOM Standard Set was implemented at two hospital sites (1 public, 1 private) in May 2016. Patients undergoing primary hip or knee replacement for osteoarthritis were recruited from pre-admission clinics and a private orthopaedic clinic. Baseline Standard Set data were collected before surgery and at pre-determined post-operative timepoints. Data on the costs of Standard Set implementation were also collected. Semi-structured interviews were conducted with key stakeholders (*n* = 15) to evaluate the ease of implementation, and explore barriers and enablers to implementation and sustainability.

**Results:**

The cost of Standard Set implementation and ongoing data collection for 17 months totalled $AUD94,955. Preference data (collected prior to completing the Standard Set) revealed that most participants preferred paper-based (83%) or web-based questionnaire completion (14%), with only a small proportion preferring iPad-based completion (3%). Several PROMs within the Standard Set were responsive to change (effect size range 0.19–0.85), with significant improvements in important health outcomes identified 6 weeks after surgery. Patient interviews showed a variable understanding of why patient-reported data collection is undertaken; however, patients perceived that PROMs provided relevant information to treating clinicians, and that the burden of questionnaire completion was minimal. Staff interviews revealed that PROMs are considered valuable, dedicated personnel are required to support data collection, gaps in information technology resources must be addressed, and that the Standard Set offers benefits beyond what currently-used measures provide.

**Conclusion:**

The Standard Set can be feasibly implemented in hospital settings, but with important caveats around staffing and technical support, consideration of patient preferences, and promotion of active clinician engagement.

**Electronic supplementary material:**

The online version of this article (10.1186/s41687-018-0062-5) contains supplementary material, which is available to authorized users.

## Background

Over the past two decades there has been a major shift towards capturing healthcare outcomes that are more patient-centred, with significant interest from clinicians, healthcare organisations and health funders. The potential value of patient-reported outcome measures (PROMs) was recently recognised by the Organisation for Economic Co-operation and Development [1]. The International Consortium for Health Outcomes Measurement (ICHOM) is a not-for-profit organisation that seeks to promote a transition to ‘value-based healthcare’ [2], which focuses on providing high-quality care and achieving optimal patient outcomes. Achieving these goals requires mechanisms for consistently capturing and reporting healthcare outcomes. ICHOM has developed standardised outcome measurement sets (termed ‘Standard Sets’) for a range of costly health conditions, and is now driving the uptake of these measurement sets in clinical practice worldwide. To date, ICHOM Standard Sets have been developed for 23 common conditions including hip and knee osteoarthritis (OA), low back pain, cardiovascular disease, stroke, and prostate cancer. The Standard Sets currently cover 54% of the global disease burden.

Hip and knee OA represent a significant international public health challenge, particularly in view of ageing populations and rising rates of obesity. The increasing burden of musculoskeletal conditions including OA is evident from the Global Burden of Disease Studies [3, 4], and is supported by data that show steady growth in the number of hip and knee replacement surgeries performed for severe OA over the last two decades [5–7]. In Australia, the number of people with OA is projected to reach 3.1 million by the year 2030 and direct healthcare costs for OA are forecast to exceed $2.9 billion by this time [8]. The growing burden of OA is also evidenced by an increase in the lifetime risk of hip and knee replacement surgeries over a 10-year period (2003–2013) as demonstrated by multi-national research [9, 10]. The ICHOM Standard Set for Hip and Knee Osteoarthritis was designed to capture outcomes of care for OA, including joint replacement surgery. This Standard Set was launched in mid-2015 and is freely available; however, until now there have been no reports of its implementation or performance in clinical settings. This study aimed to:

- implement the ICHOM Standard Set for Hip and Knee Osteoarthritis for patients undergoing joint replacement surgery for OA in public and private hospital settings;
- evaluate the feasibility and costs of implementation; and
- explore stakeholder experiences (patients, clinicians and administrative staff) regarding the ease of implementation and use of the Standard Set in these settings.

## Methods

### Study design

A mixed-methods design was used to capture comprehensive data on patient outcomes, implementation costs, and the implementation experiences of patients, clinicians and administrative staff.

### The ICHOM standard set for hip and knee osteoarthritis

Each ICHOM Standard Set is developed using a consensus-based process involving extensive consultation with experienced clinicians, measurement researchers and patient representatives. The Standard Sets incorporate existing PROMs instruments and new measurement items. They are designed to cover the full cycle of patient care (comprising non-surgical and surgical treatment) and can be used across different healthcare settings. The Standard Sets represent a minimum dataset (users are free to collect additional variables) and there are minimum recommended time points for data collection (additional time points can be added). The Standard Sets are freely available, although some of the PROMs measures contained within may require a license for use. The Standard Set can be obtained from the ICHOM website (http://www.ichom.org/medical-conditions/hip-knee-osteoarthritis/).

**Fig. 1:**
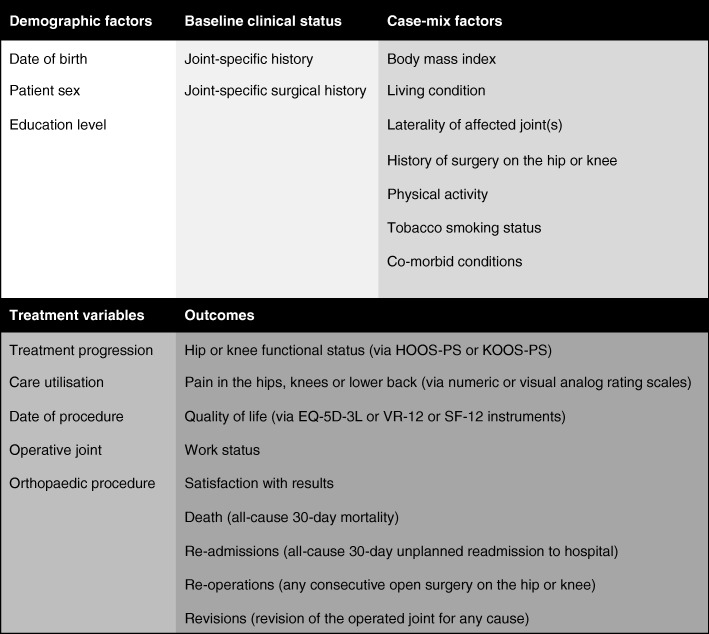
Overview of ICHOM Standard Set for Hip and Knee Osteoarthritis variables EQ-5D-3L: EuroQoL 5-dimension health-related quality of life instrument (3 response level version); HOOS-PS: Hip Disability and Osteoarthritis Outcome Score - Physical function short form; KOOS-PS: Knee Injury and Osteoarthritis Outcome Score - Physical function short form; SF-12: Short Form 12 Health Survey; VR-12: Veterans RAND 12 instrument

Development of the Standard Set for Hip and Knee Osteoarthritis commenced in July 2014 and the completed Standard Set was launched in July 2015 [11]. The international Working Group that developed the Standard Set for Hip and Knee Osteoarthritis comprised joint replacement registry leaders, orthopaedic surgeons, rheumatologists, physiotherapists and patients with OA from 10 countries [12]. The Standard Set for Hip and Knee Osteoarthritis contains a comprehensive set of variables (Fig. 1) including demographic factors, clinical status, case-mix factors, treatment variables, patient-reported health status (self-reported functional, pain, and quality of life outcomes) as well as all-cause mortality, re-admissions and re-operations.

### Study setting

Australia has parallel public and private health systems, with 59% of hip replacements and 71% of knee replacements performed within the Australian private hospital system [7]. It is therefore essential that any research undertaken in this field considers public and private hospital settings, in order to provide a comprehensive perspective. This study was conducted in Melbourne, Australia at The Royal Melbourne Hospital (RMH) and at the private consulting rooms of an orthopaedic surgeon at the Melbourne Private Hospital (MPH). The RMH is a major tertiary public hospital with over 450 beds. It has a large orthopaedic surgery department, comprising 16 orthopaedic surgeons. It performs approximately 200 primary and revision hip and knee replacement surgeries each year and over 600 appointments are offered annually in the outpatient OA clinics. It is co-located with a private hospital (MPH), where privately-insured patients are seen in individual orthopaedic surgeon consulting rooms.

### Participants

Study participants comprised patients and other key stakeholders involved in the assessment or management of patients undergoing hip or knee replacement surgery for OA.

#### Patients

For the quantitative component of the study, English-speaking patients undergoing primary elective hip replacement or primary knee replacement surgery for OA were eligible to participate in the study. Non-English speaking patients were also eligible to participate, provided they had a proxy available to assist them in completing the English language questionnaires. It was not possible to translate the ICHOM Standard Set into multiple languages, given the broad cultural composition of the hospitals’ catchment area. All patients undergoing primary hip or knee replacement surgery for OA were approached and screened for eligibility. The project co-ordinator oversaw the recruitment of consecutive, eligible patients from the pre-admission clinics (PAC) at RMH and follow-up of study participants via the post-operative Orthopaedic Outpatient Clinics and Joint Replacement Surgery Clinics. Patients presenting to a private orthopaedic clinic at MPH for hip or knee replacement surgery for OA were also recruited and followed up by the project co-ordinator, after notification of upcoming appointments by the orthopaedic surgeon or their practice manager.

Eligible patients were recruited within 3 months prior to their scheduled surgery date. Recruitment was undertaken at the RMH PAC appointment or the relevant orthopaedic consultation at MPH. The recruitment process was conducted by a senior musculoskeletal physiotherapist working in the PAC with support from the project co-ordinator. All eligible patients were provided with detailed information about the project and advised that completion of the study questionnaires would constitute implied consent, consistent with our ethics approval. All recruitment for the study was undertaken between May 2016 and June 2017.

For the qualitative component, patients with personal experience of undergoing joint replacement surgery were also recruited for individual semi-structured interviews. As the interview questions largely related to completing questionnaires before and after surgery, it was considered that these questions would not be relevant to patients who had not yet received joint replacement surgery. Patients were purposely sampled from the overall cohort to incorporate a range of demographic characteristics, for example, gender, age group and hip versus knee replacement surgery. Interview participants were provided with a Participant Information and Consent form, and written informed consent for the interview was obtained from each individual.

#### Other key stakeholders

The project co-ordinator also recruited orthopaedic surgeons, musculoskeletal physiotherapists, hospital executive staff, and clinic administrative staff from the clinical sites to participate in stakeholder interviews. All interview participants were provided with a Participant Information and Consent form, and written informed consent was obtained from these individuals.

### Data collection

#### Quantitative data

The ICHOM Standard Set was implemented in May 2016, with data collection continuing until September 2017. Pre-implementation education was provided on a one-on-one, informal basis by the study co-ordinator to relevant individuals (for example, clinic physiotherapists and orthopaedic surgeons) prior to implementation of the Standard Set. This education included information on the Standard Set and the processes for patient recruitment and administering the Standard Set pre- and post-operatively. Quantitative data collection included patient outcomes from joint replacement surgery (through the collection of ICHOM Standard Set for Hip and Knee Osteoarthritis variables) as well as implementation costs. The Standard Set offers three generic (non-disease-specific) alternatives for assessing health-related quality of life; the EQ-5D-3L instrument was selected for this study and a research license was obtained from EuroQol.

Baseline data collection was undertaken prior to surgery (timed with the pre-admission clinic visit, usually within 6 weeks prior to the scheduled surgery date). At this time the ICHOM Standard Set for Hip and Knee Osteoarthritis was administered, and the patient’s preference for iPad, paper-based or web-based questionnaire completion was recorded prior to completing the Standard Set. All required baseline clinical data were extracted manually from the relevant databases. Follow-up data collection was undertaken at 6 weeks, 3 months, 6 months and 12 months post-operatively. At these time points, the ICHOM Standard Set was re-administered by mail and manual extraction of required clinical data was performed by the project co-ordinator. Data from the 3 month, 6 month and 12 month time points are not presented in this paper as the study was designed to assess the feasibility of Standard Set implementation rather than evaluate the effectiveness of joint replacement surgery over time (the latter is already well established in the literature). Data on patient deaths or all-cause hospital readmissions within 30 days of surgery, and re-operation or revision joint replacement surgery were extracted from existing hospital databases.

It was anticipated that the PROMs would be administered in a variety of formats, depending on patient preferences and available resources (e.g. paper-based questionnaires or questionnaires completed using portable electronic devices in clinics, and mail-based or internet-based formats for home administration). Both iPad and paper-based formats were trialled in this project, and the iPad interface was pilot tested for approximately 2 months prior to Standard Set implementation, to refine the design and optimise functionality. Initially both the iPad and paper-based data collection formats were made concurrently available (depending on patient preference); however, from June 2016 onwards only paper-based questionnaires were used (given pragmatic challenges associated with iPad use and data extraction, and in view of patient preference data) while a portal for web-based questionnaire administration was being developed. Although the web-based portal was developed, it was unfortunately not able to be pilot tested or implemented during this feasibility study due to a lack of dedicated IT support.

Any non-completed questionnaires and missing item responses were followed up in a timely manner (usually within 1 week) by the project co-ordinator. Where there were few missing item responses, participants were telephoned and asked to provide their responses over the phone. Where there were many missing item responses, participants were sent a copy of their semi-completed questionnaire with missing responses highlighted for their completion and return. Where questionnaires were not returned, participants were initially contacted by phone as a reminder and subsequently by mail, to maximise response rates.

Data on the costs of Standard Set implementation and ongoing data collection were collected during the study period. These included costs relating to project management by the project co-ordinator, IT support, external implementation support from ICHOM, staffing support from other physiotherapists who assisted with patient recruitment and data collection, and the cost of postage and reply-paid postage for study questionnaires. The project co-ordinator recorded the hours spent on specific study tasks (for example, administrative tasks, IT-related tasks, handover and other meetings, and patient recruitment). All costs are reported in Australian dollars (1 AUD = 0.75 USD).

#### Qualitative data

Fifteen individual semi-structured, face-to-face or telephone interviews were conducted with key stakeholders (8 patients, 7 staff members) to evaluate the ease of Standard Set implementation, and explore barriers and enablers to successful implementation and future sustainability. The study team developed the interview schedules including prompt questions (Additional file 1). All qualitative data collection was conducted by BC between May and August 2017. The interviews were electronically recorded to facilitate verbatim transcription of the data. Each patient interview was, on average, 6 min in duration and the other stakeholder interviews were, on average, 12 min in duration.

### Data analysis

#### Quantitative data

Demographic, clinical, case-mix and outcomes data were analysed descriptively using SPSS Statistics v23. EQ-5D index scores were calculated using published Australian preference weights [13]. HOOS and KOOS physical function subscale scores (HOOS-PS and KOOS-PS scores, respectively) were transformed to a 0–100 scale using the relevant nomogram from the developer’s website (www.koos.nu). The ICHOM instruments were scored as outlined below:

- All pain numeric rating scales (NRS): 0 (no pain at all) to 10 (worst pain imaginable)
- EQ-5D index: 0 (death) to 1 (full health), with negative scores indicating a health state worse than death
- EQ-5D Visual Analogue Scale (VAS): 0 (worst health state) to 100 (best health state)
- HOOS-PS: 0 (best hip-related function) to 100 (worst hip-related function)
- KOOS-PS: 0 (best knee-related function) to 100 (worst knee-related function).

The feasibility of assessing patient outcomes using the Standard Set was specifically evaluated by examining the proportion of missing data and responsiveness to change. The proportion of missing baseline data for each patient-reported item of the Standard Set was calculated. The responsiveness of the Standard Set PROMs (reflecting the ability of the instruments to detect change over time) was evaluated by calculating effect sizes and relative efficiency. The 6-week time point was used for the responsiveness analyses, as this was the first post-operative assessment. Effect sizes were calculated by dividing the change score (difference between the mean baseline and 6-week scores) for each measure by its baseline standard deviation [14] for all participants who provided baseline and 6-week data. Effect sizes were categorised into small (0.20–0.49), medium (0.50–0.79) or large (≥0.80), according to Cohen’s classification [15]. Paired t-tests were used to determine change in key outcomes from baseline to 6 weeks for the purpose of calculating relative efficiency [16]. The t-score was squared (t^2^) and the instrument with the highest t^2^ value was used as the reference (ascribed a relative efficiency of 1.00). Relative efficiency was calculated by dividing the t^2^ value for each measure by the t^2^ value of the reference.

Costs data were analysed using Microsoft Excel 2013; the costs relating to implementation planning, implementation, external ICHOM support, and 17 months of Standard Set data collection were considered. The costs analysis excluded time spent on qualitative interviews, data extraction, data analysis, and report preparation as these relate largely to the study evaluation. Staffing costs were calculated based on current Victorian annual salaries for employed staff, including on-costs. The database manager costs were calculated using the ‘Scientist, Grade 3, Year 4’ classification (annual salary of $115,506), the project co-ordinator costs were calculated using the ‘Senior Clinician Physiotherapist, Year 4’ classification (annual salary of $134,030), and additional musculoskeletal physiotherapist costs were calculated using the ‘Senior Clinician Physiotherapist, Year 2’ classification (annual salary of $121,777).

#### Qualitative data

Interview data were analysed using QSR NVivo10, a software package designed to support thematic analysis. Thematic analysis was predominantly undertaken by BC, who has previous experience in qualitative data analysis. An inductive approach [17] was used to identify and code key themes arising from the interview transcripts until no new themes emerged. A second reviewer (INA) examined each transcript to confirm and refine the themes identified. Any discrepancies were discussed and resolved by consensus. Verbatim responses to questions were de-identified for reporting purposes and selected responses are provided to illustrate emergent themes.

## Results

### Flow of participants through the study

**Fig. 2:**
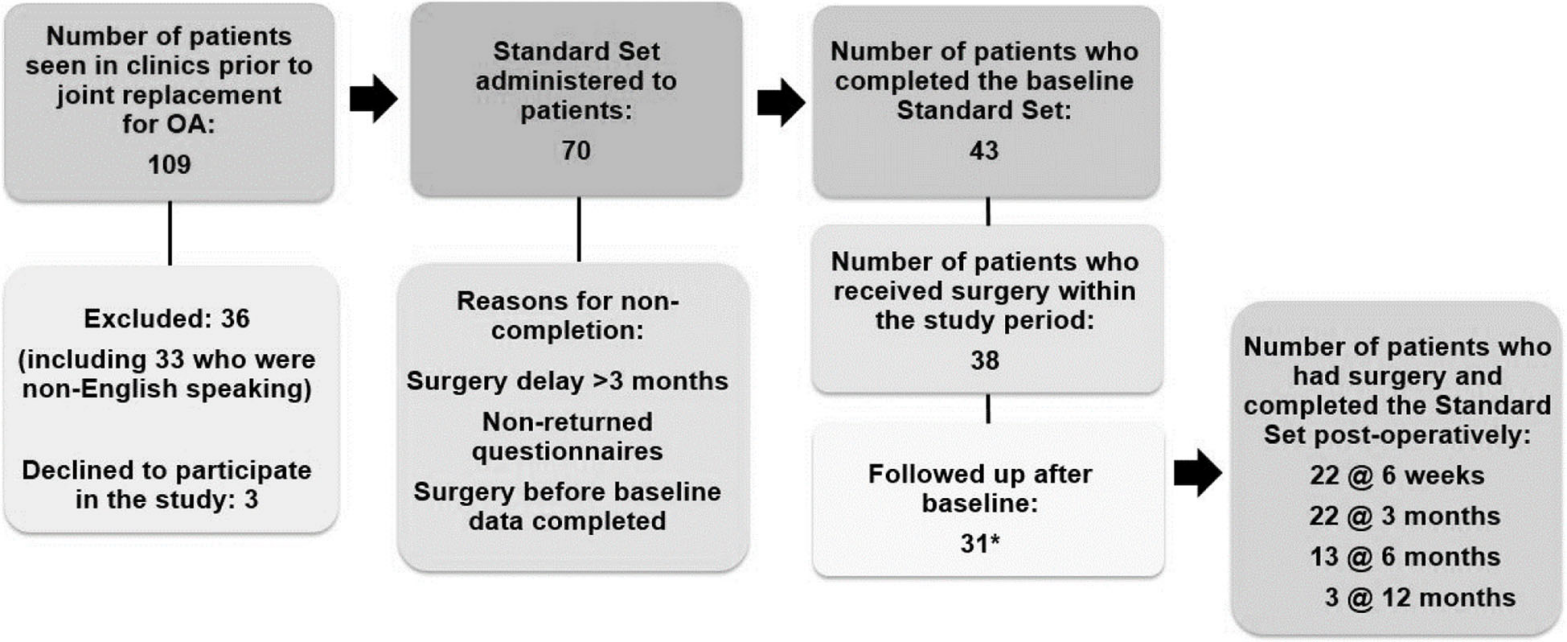
Flow of study participants. * 7 participants were not followed up beyond baseline for the following reasons: delay of > 3 months between baseline and their surgery (*n* = 5); evident post-operative cognitive impairment (*n* = 2)

The flow of study participants is summarised in Fig. 2. In total, 109 patients were seen prior to joint replacement surgery for OA over the study period. Of these, 36 patients were excluded as they did not meet the eligibility criteria. This included 33 patients who were non-English-speaking, 2 patients with cognitive impairment and 1 patient who had rheumatoid arthritis. A further 3 patients declined to participate in the study due to disinterest (*n* = 2) or limited available time (*n* = 1). The baseline Standard Set was administered to 70 participants and of these, 43 completed the Standard Set (61% response rate). Reasons for non-completion included a surgery delay of more than 3 months (reflecting ICHOM guidelines that baseline data be collected within 3 months prior to surgery), non-returned questionnaires, or surgery being performed before baseline data were collected. The majority of participants were recruited from RMH (*n* = 35, 81%) and the remainder were recruited from the private clinic. Use of a proxy to complete the baseline questionnaire was very infrequent (*n* = 1) and the reason for this assistance is not known (this could include, but not be limited to, English language limitations, low vision and general literacy).

Of the participants who completed a baseline questionnaire, 38 received joint replacement surgery within the study period. However, 5 participants had a delay of more than 3 months between completing a baseline questionnaire and their subsequent surgery and were not followed up beyond baseline. Of the 33 remaining participants, 2 participants had evident post-operative cognitive impairment and were not followed up further. Of the remaining 31 participants, 22 completed the Standard Set at 6 weeks (71% of those who reached this time point in the study; 3 questionnaires were not returned and 6 were missed due to protracted staff leave), 22 completed the Standard Set at 3 months (76% of those who reached this time point; 6 questionnaires were not returned and 1 was missed), 13 completed the Standard Set at 6 months (72% of those who reached this time point; 4 questionnaires were not returned and 1 was missed) and 3 completed the Standard Set at 12 months (100% of those who reached this time point). A number of participants had not reached their 6 month and 12 month post-operative time points by the time the study concluded.

### Participant preferences

All patients approached to complete the baseline Standard Set (*n* = 70) were asked about their preference for paper-based, web-based or iPad-based questionnaire completion. Patient preference data revealed strongly that most participants preferred paper-based questionnaire completion (*n* = 52, 83% of those who responded), while few preferred web-based (*n* = 9, 14%) or iPad-based data collection (*n* = 2, 3%).1

### Participant characteristics

On average, baseline questionnaires were completed within 6 weeks prior to surgery (median 41 days, interquartile range 19–83 days). Table 1 summarises the demographic characteristics of the study participants at baseline. The average age of participants at baseline was 69 years (interquartile range (IQR) 66–73 years) and there was a similar proportion of males and females. Most participants had attended primary or secondary school (76%), although few had attained a tertiary education (21%). Eighty-eight % of participants were overweight or obese. Although the majority were not working by choice or seeking employment (62%), 17% of participants reported that they were unable to work due to their OA. Doctor-diagnosed co-morbidities were common, with hypertension (63%) and conditions affecting the spine (58%) most frequently reported.

**Table 1:** Demographic characteristics

Characteristic (*n* = 43)	
Median age, years (IQR)	69 (66–73)
Gender, *n* (%)	
Male	22 (51)
Female	21 (49)
Highest level of education, *n* (%)	
Primary or secondary school	32 (76)
Tertiary	9 (21)
Body Mass Index category, *n* (%)	
Underweight / normal weight	5 (13)
Overweight	17 (43)
Obese	18 (45)
Living arrangement, *n* (%)	
Lives with partner/family/friends	31 (74)
Lives alone	10 (24)
Other	1 (2)
Work status, n (%)	
Unable to work due to a condition other than osteoarthritis	3 (7)
Unable to work due to osteoarthritis	7 (17)
Not working by choice or seeking employment	26 (62)
Working (either part-time or full-time)	6 (14)
Current smoker, *n* (%)	5 (12)
Doctor-diagnosed co-morbid conditions, *n* (%^a^)	
High blood pressure	27 (63)
Arthritis in the back or other spine condition	25 (58)
Diabetes	10 (23)
Heart disease	9 (21)
Lung disease	9 (21)
Depression	9 (21)
Rheumatoid arthritis or other arthritis (in addition to OA)	9 (21)
Leg pain when walking due to poor circulation	7 (16)
Cancer within the last 5 years	5 (12)
Problems caused by a stroke	2 (5)

Numbers may not total 43 due to missing responses to individual items

^a^Total exceeds 100% as some participants reported more than 1 co-morbid condition

### Performance of the ICHOM Standard Set measures

#### Missing item responses

The high level of data completeness at baseline reflects the strong commitment to follow up of missing data by the project co-ordinator. Information on the quantity of missing data prior to follow-up was not systematically recorded; however, all missing item responses were followed up by the project co-ordinator during the study period, apart from periods of staff leave. There were some instances where the participant could not be contacted or did not complete the missing items, although this specific information was not recorded. The ultimately low proportion of missing responses indicates that the ICHOM-recommended patient-reported items were well-tolerated by participants across the demographic, disease-related, pain, EQ-5D, KOOS and HOOS questions (Table 2). Four participants (9%) did not respond to the question regarding treatment satisfaction at baseline; this item may have been confusing (it allows patients to reflect on any OA-related treatment received) given that they had not received their joint replacement surgery as yet.

**Table 2:** Missing self-reported ICHOM Standard Set data at baseline

Variable (*n* = 43, except where otherwise specified)	Number of participants who did not respond (%)
Demographic variables (*n* = 43)	
Sex	1 (2)
Education level	1 (2)
Height	3 (7)
Weight	2 (5)
Living arrangement	1 (2)
Work status	1 (2)
Smoking status	1 (2)
Physical activity	1 (2)
Disease-related variables (*n* = 43)	
Doctor-diagnosed self-reported osteoarthritis	2 (5)
Previous surgery for osteoarthritis	1 (2)
Treatments for osteoarthritis in the past 6 months	1 (2)
Healthcare providers seen for osteoarthritis treatment in the past 6 months	2 (5)
Satisfaction with the results of treatment	4 (9)
Pain variables (*n* = 43)	
Pain - right hip	3 (7)
Pain - left hip	3 (7)
Pain - right knee	2 (5)
Pain - left knee	2 (5)
Pain - lower back	2 (5)
EQ-5D items (*n* = 43)	
Mobility item	2 (5)
Self-care item	2 (5)
Activity item	3 (7)
Pain / discomfort item	2 (5)
Anxiety / depression item	2 (5)
Visual analogue scale	2 (5)
HOOS-PS items (hip participants only, *n* = 8)	
Descending stairs	0 (0)
Getting in / out of bath or shower	0 (0)
Sitting	0 (0)
Running	0 (0)
Twisting / pivoting on your loaded leg	1 (13)
KOOS-PS items (knee participants only, *n* = 35)	
Rising from bed	1 (3)
Putting on socks / stockings	1 (3)
Rising from sitting	1 (3)
Bending to floor	1 (3)
Twisting / pivoting on injured knee	1 (3)
Kneeling	1 (3)
Squatting	1 (3)

#### Responsiveness of the ICHOM Standard Set measures

Significant improvements in important health outcomes were identified as early as 6 weeks after surgery, supporting the responsiveness of specific PROMs tools. When examining changes in health outcomes from baseline to 6-week follow-up, only the EQ-5D index demonstrated a large effect size. This indicates that despite our use of the 3-level EQ-5D instrument (with 3 response options per item, compared to the more sensitive 5-level instrument that is now available), this measure is still capable of detecting improvements after joint replacement surgery. Medium effect sizes were found for the knee and lower back pain NRS items, and for the KOOS physical function scale. Only small effect sizes were found for the hip pain NRS items and the EQ-5D VAS, and these post-operative changes were not statistically significant. The relative efficiency statistic was used to rank the ICHOM Standard Set health outcome instruments according to their responsiveness to change (Table 3). According to this statistic, the pain in right knee NRS was the instrument that was most responsive to change from baseline to 6 weeks (relative efficiency of 1.00), followed by the pain in left knee NRS (relative efficiency of 0.82) and the EQ-5D index score (relative efficiency of 0.59). Interestingly, the disease-specific KOOS instrument was less responsive to change than the generic EQ-5D index (relative efficiency of 0.35 vs 0.59).

**Table 3:** Effect sizes from baseline to 6 weeks (*n* = 22) and relative efficiency

Instrument	Mean change^a^ (95%CI)	p	Effect size	Relative efficiency^b^	Relative efficiency rank
Pain - right knee	−2.67 (−4.10 to −1.23)	< 0.01	0.79 (medium)	1.00	1
Pain - left knee	−1.78 (− 2.84 to −0.72)	< 0.01	0.51 (medium)	0.82	2
EQ-5D index	0.23 (0.07 to 0.39)	0.01	0.85 (large)	0.59	3
Pain - lower back	−1.89 (−3.25 to −0.53)	0.01	0.77 (medium)	0.56	4
KOOS physical function^c^	−9.87 (−19.19 to −0.55)	0.04	0.74 (medium)	0.35	5
Pain - right hip	−1.17 (−2.83 to 0.50)	0.16	0.39 (small)	0.14	6
EQ-5D VAS	5.53 (−4.52 to 15.57)	0.26	0.26 (small)	0.09	7
Pain - left hip	−0.44 (−1.78 to 0.89)	0.49	0.19 (small)	0.03	8

^a^Mean change = 6-week post-operative score minus baseline score; positive change represents improvement for the EQ-5D index and EQ-5D VAS; negative change represents improvement for pain scores, and KOOS-PS score; *p* < 0.05 indicates a statistically significant change

^b^Relative efficiency < 1.00 indicates that the instrument is less efficient than the ‘Pain - right knee’ score in detecting change after joint replacement surgery

^c^KOOS physical function scores collected from knee participants only; HOOS physical function scores are not reported as too few hip participants completed both baseline and 6 week questionnaires (*n* = 3)

#### Implementation costs

The costs of implementation and 17 months of Standard Set data collection (May 2016 to August 2017) totalled $94,955. This included the following cost components:

- Project co-ordinator time: $46,846.

This included time spent on administrative tasks, ethics applications and amendments, IT liaison and IT-related tasks, database management and direct patient contact for recruitment, data collection and follow-up of missing data

- IT support: $25,989.

This included time spent on the development of the iPad and web-based data collection interfaces and database development, as well as the development of reporting capabilities

- ICHOM implementation support: $19,215.

This includes a site visit to Melbourne, regular teleconferences and email-based support

- Equipment and consumables: $1846.

This includes the estimated costs of iPads for use in clinics and actual postage costs for questionnaires (both initial and reply-paid postage)

- Pre-admission clinic physiotherapist: $1059.

This includes time spent on administrative tasks and direct patient contact for recruitment and data collection (including during periods of project co-ordinator leave).

### Key interview themes - patient interviews

While the patient interviews were relatively short in duration, they elicited four key themes, as summarised below.

#### Key theme 1: Patients’ understanding of patient-reported data collection is variable

Interview participants expressed a range of views regarding why pre- and post-operative questionnaires are used (*n* = 7). This emphasises that additional education may need to be provided to patients to help them understand why PROMs data are being collected and how the tools used differ from a patient experience survey. Some of the responses received included:

*“Just so that you can establish um the healing process and sort of what happens after you leave the hospital and any problems that may arise after surgery.”* (Patient 3).

*“To estimate whether there’s procedures in place that are working properly.”* (Patient 5).

*“To help improve treatments, I suppose.”* (Patient 7).

*“To me, it’s um to improve on hospital procedures. You know exactly how people are going about things and what people think. Public relations. Put it that way.”* (Patient 8).

#### Key theme 2: PROMs are perceived to be valuable

Some interview participants (*n* = 4) perceived that completion of PROMs instruments was valuable for providing hospital staff with information regarding the patient’s wellbeing.

*“…it’s just beneficial to everybody, for people like myself to fill in these forms because it gives everybody an understanding of what we’re going through, you know.”* (Patient 2).

*“Yes because they can get an understanding of how the patient actually feels.”* (Patient 5).

*“Well I think it gives you an input into what pain we’ve got and hopefully treatment to fix it.”* (Patient 7).

Some interview participants (*n* = 3) also perceived that the completion of PROMs assisted clinicians to evaluate the outcomes of surgery and the recovery process.

*“Oh yes, most definitely, because the doctors can go back on that and have a look to see how you went after the operation, with the difference before and after.”* (Patient 1).

Several interview participants (*n* = 5) also perceived a direct personal benefit to themselves as a result of completing the PROMs instruments.

*“It is inclusive too, I think if you have surveys done before you have the surgery and after you have the surgery you feel as though you’re not alone and you’ve got some other body sort of interested in your sort of recovery, and preparation.”* (Patient 3).

*“Yeah because I looked back over the original one and then I looked at the current ones to see how I’d progressed and it gives me an idea that I’m heading in the right direction.”* (Patient 6).

#### Key theme 3: Completion of PROMs represents a minimal burden on patients

There was strong agreement among interview participants (*n* = 8) that the time required to complete the PROMs was minimal and did not impose a burden on patients.

*“No problem whatsoever.”* (Patient 2).

*“Look, I don’t think it was an enormous amount of time.”* (Patient 4).

*“Ah, it only took about 5 minutes. There was no inconvenience to it.”* (Patient 8).

#### Key theme 4: Paper-based questionnaire completion is preferred

Consistent with our quantitative findings, interview participants (*n* = 7) reported a strong predilection for paper-based questionnaires, particularly if sent to them at home, rather than alternative modes of questionnaire completion. Two participants reported they did not have internet access, so would be unable to complete web-based questionnaires at home. Interestingly, no concerns were raised about the potential burden of paper-based questionnaire collection on hospital staff.

*“…that would be fine but I don’t have the internet, but if you sent it home to me I’d be able to do it.”* (Patient 1).

*“…well it doesn’t matter whether it’s in clinic or to home, I’m not online so it would have to be that way.”* (Patient 2).

*“I’d rather the paper sent to the home, thank you.”* (Patient 3).

*“Sent to the house I think is easier, for everyone.” (Patient 7).*

### Key interview themes - other stakeholders

Four key themes were identified from the staff interviews, as described below.

#### Key theme 1: PROMs are perceived to be valuable

There was general consensus that PROMs collection is considered valuable (*n* = 7). Some interview participants spoke about PROMs as a useful tool for facilitating and guiding clinical conversations between the patient and clinician (*n* = 4) and about the role of PROMs in the provision of patient-centered care (*n* = 2):

*“I think it’s positive and contemporary practice and supports meaningful consumer engagement and participation in their care, and can inform the development and change of practice, if needed.”* (Non-clinical professional 1).

*“It definitely supports that collaboration in decision making and informing new ways of working.”* (Non-clinical professional 1).

*“I think for patients it’s a useful communication tool for communicating with their clinicians and it is, I think patients find it reassuring that we are monitoring their outcomes.”* (Orthopaedic surgeon 1).

*“It’s an adjunct to our clinical conversation, but not a replacement for.”* (Orthopaedic surgeon 2).

*“So for clinicians it allows us to understand someone’s condition better…It also prompts us to ask questions which are about people’s life that we may not otherwise do.”* (Physiotherapist 1).

Interview participants also described the ways in which PROMs data can be utilised in clinical practice; for example, for assessing the outcomes of surgery (*n* = 7), to monitor patient progress (*n* = 3) throughout the continuum of care, and to support the prioritisation of patients (for example, those on surgical waiting lists) (*n* = 2).

*“I think they’re a good way of tracking how someone has changed before and after their surgery.”* (Physiotherapist 1).

*“I think they’re valuable, because it gives you an idea of whether the patient has improved or not improved, both pre- and post-operatively.”* (Orthopaedic surgeon 2).

*“It helps you to monitor the patient’s progress and to pick up patients in whom there is a problem or deterioration.”* (Orthopaedic surgeon 1).

*“…and then there’s obviously the tracking of patient recovery with time.”* (Orthopaedic surgeon 2).

*“I think it’s essential that we monitor our outcomes, for all surgery… and I think with hip and knee replacements the most useful or the best outcome measure we have to date is patient-reported outcome measures.”* (Orthopaedic surgeon 1).

*“…using them beforehand can help identify people who maybe need to be prioritised higher or lower.”* (Physiotherapist 1).

Several interview participants also perceived that PROMs were of value in effectively communicating the outcomes of surgery to patients (*n* = 3).

*“And I think that actually helps the patient too, I think it reminds the patient that they’re, what they were like and then a graph or a series of numbers can clearly show it to say ‘look you were here, now you’re here’, ‘you were low, and now you’re high and you’ve clearly improved.”* (Orthopaedic surgeon 2).

*“I think for patients as I said, sometimes they forget, you know, maybe how poor they were functioning pre-operatively and when they feel that they’ve got prolonged pain post-operatively they may not think that the surgery was worthwhile and they have made clinical improvements, but having this outcome measure done pre- and post-op can show them that it was worthwhile and they didn’t undergo something for nothing, show them there was a change.”* (Physiotherapist 2).

*“…it’s a good way of then showing them to say ‘look, from before surgery you were at this level, now you’re at that level’, so it’s one tool in which we can then have a clinical conversation with the patient.”* (Physiotherapist 3).

#### Key theme 2: The requirement for dedicated personnel

Interview participants described the challenges associated with PROMs co-ordination, including the difficulties associated with managing paper-based questionnaire administration in view of time constraints (*n* = 4) within the busy outpatient clinic setting.

*“It takes a lot of admin process to give it out, collect it, collate it.”* (Physiotherapist 3).

*“…I think the difficulty is the actual logistics of administering it in a clinic that’s time poor.”* (Orthopaedic surgeon 2).

*“You know you’ve got a busy clinic with 20 patients waiting, sort of the PROMs gets, it’s the first thing that gets cut when you’re stuck for time.”* (Orthopaedic surgeon 2).

*“…if you wanted it completed in clinic there would need to be extra time set aside for that...”* (Physiotherapist 2).

There was consensus among participants (*n* = 6) that dedicated staffing support would be required to ensure future sustainability of PROMs collection. It was evident that future data collection (beyond the implementation study) would be challenging without appropriate staff resources.

*“Well, it definitely needs dedicated resource to do it properly, it’s very challenging, tricky work and it can, yes, if we get the technology right it can streamline it but you still need someone to be the face of it, to lead it, to drive it, to do the analysis, to write reports, to deliver presentations about what the findings are, to make the changes and do all the change management around that so it’s not just about capturing data, it’s about the outcomes and use of that data for information for change.”* (Non-clinical professional 1).

*“Sustainability, it can be very sustainable provided there’s enough support to enable that to happen… So it should be sustainable, it can be sustainable, based on resources. I think the clinicians’ intentions are there and we recognise that and we know the potential benefit but the day-to-day barrier of clinical care can get in the way so if we are going to make it sustainable, which we feel we should, then we are just going to need resources and support to help that.”* (Physiotherapist 3).

#### Key theme 3: Identified gaps in IT resources

The limited availability of IT infrastructure to support PROMs collection beyond the study period was highlighted by some participants (*n* = 2), with emphasis that investment in this area is required to ensure sustainability of future data collection.

*“We don’t have the IT resources to do it and I’m trying to completely you know transform that barrier….”* (Orthopaedic surgeon 1).

*“Well I think we know that there’s been problems around the data capture and the platform that we’ve been using so I think it’s a concern around sustainability that we’re adequately resourced and we’ve got an adequate platform to use in the future and that has obviously become more true over time, that we’ve had some difficulties with that.”* (Non-clinical professional 1).

*“The IT resources and time commitment are big barriers, busy clinicians, and the one can solve the other to some extent because if you can administer the PROMs efficiently with using IT rather than using up clinician time that would improve uptake significantly and I think also that the IT could be used to identify patients that should be doing PROMs and do them even without any clinician input, that would be the gold standard I think we should be aiming for.”* (Orthopaedic surgeon 1).

*“Well we need an IT system to roll out and collect the scores from patients over the internet and we need somebody to run that database, maintain that database, whether that be internal or external but either way there’s a cost associated with it.”* (Orthopaedic surgeon 1).

#### Key theme 4: Benefits associated with using the ICHOM standard set

Interview participants perceived a range of benefits associated with using the ICHOM Standard Set for Hip and Knee Osteoarthritis, beyond what currently-used measures provide. There was perceived value in using a standardised approach to enable the benchmarking of joint replacement outcomes (*n* = 4). The Standard Set was perceived by some interview participants as being evidence-based, expert-derived and robust (*n* = 3), with recognition of ICHOM’s international standing.

*“And by having a standard set of data that could be utilised, it means that better cross-institution research could be done, better comparisons with other institutes could be done, and performance between countries could even be then more easily compared.”* (Orthopaedic surgeon 2).

*“Just to set up this process in a robust way that was probably, the strength was in the ICHOM, you know the validity of the dataset and the robustness of the evidence base, and having that sort of international collaborative behind it, so that felt stronger than us just trying to use a sort of random quality of life rating.”* (Non-clinical professional 1).

*“It’s also a dataset which is used nationally and internationally as compared to a survey that we developed ourselves and haven’t really validated so I feel like it’s a much more rigorous process compared to the ones I’ve been previously involved in… It feels like it probably has more value in the long term rather than something we’re doing in isolation.”* (Physiotherapist 1).

*“…the ICHOM Standard does seem to be, again I like the fact that it’s evidence-based, it’s comprehensive, it looks at different domains and it’s striving for consistency amongst the health organisations which is a real key, cause there’s no point different centres doing different things, so the idea of standardisation of evidence base is really appealing.”* (Physiotherapist 3).

However, some interview participants did raise specific concerns about implementing and using the ICHOM Standard Set in relation to its content and length (*n* = 2).

*“One of the barriers to implementation of the Standard Set is that it’s different to the measures that most hospitals are using at the moment and so it requires a change and it requires either to use the Standard Set in addition to the existing measures they’re using or to then change the measures they’re measuring which will mean that we abandon the data that they’ve already collected essentially.”* (Orthopaedic surgeon 1).

*“The ICHOM scores are a lot more than what I have been collecting historically in that I usually use, I’ve been using the MAPT and Oxford scores for all my patients and the ICHOM scores are much more involved than that and I’m not sure that my practice manager has time, or my patients have time, to fill in all those except on special occasions.”* (Orthopaedic surgeon 1).

*“The only other difference along that same line, because it is more detailed, it probably takes longer to administer or complete.”* (Physiotherapist 2).

Of note, interview participants largely equated ICHOM Standard Set data collection with PROMs collection. The collection of other variables within the Standard Set (for example, clinical, mortality, and readmission data) was not specifically mentioned by interview participants.

## Discussion

It is increasingly recognised that PROMs can be used in a variety of ways: to support clinical care [18], to guide healthcare funding and resource allocation decisions [19, 20], and for benchmarking [21, 22]. Supporting these functions, the ICHOM Standard Sets provide a readily available and internationally-recognised mechanism for longitudinal data collection and a means for reporting healthcare outcomes to clinicians, healthcare organisations and health funders. However, we have a limited understanding of how the Standard Sets perform in clinical settings as few implementation reports exist [23, 24]. Our study therefore provides important information on implementation feasibility (and in particular, the costs of implementation) as well as factors impacting the sustainability of Standard Set data collection. We are keen to share our learnings to support others who are considering implementing the Standard Sets in their own institutions.

Based on our experiences, the ICHOM Standard Set for Hip and Knee Osteoarthritis can be feasibly implemented in ‘real world’ clinical settings, but with a number of important caveats. Firstly, it is clear that implementation and ongoing data collection requires a dedicated person to manage all of the required processes. We consider this role is best suited to a clinician who is familiar with existing hospital databases and already has a direct patient contact role. In our experience, staffing at 0.2 to 0.4 full-time equivalent was required, with additional weekly support provided by other clinical staff. Adequate staffing support is essential to support the implementation of PROMs tools, facilitate ongoing data collection, and maximise the completeness of data collection.

Secondly, strong IT support is essential for database development, the development of patient and clinician reminder systems and data collection interfaces, and data extraction tasks. As access to hospital IT support may not suffice (or may not be available), funding for IT support should be built into the design of any future Standard Set implementation work. The value of a high-quality IT platform was also emphasised in a case study describing the implementation of the ICHOM Standard Set for Cleft Lip and Palate in the Netherlands [23]. Ideally, ‘real-time reporting’ for clinicians will be most valuable, so that clinicians have access to patient scores at the point of care and can use this information to guide shared decision-making and plan clinical care.

Thirdly, active clinical engagement is essential for sustainability and needs to start early so clinicians can understand the potential worth of collecting Standard Set data and how these data might be used (e.g. for patient review purposes, planning future care, or benchmarking patient outcomes between hospitals, states and countries). We invested significant time into engagement activities across multiple levels (including at clinician, hospital management and state government levels) over the study period. The education of staff and patients, and the facilitation of staff culture change have been identified as key lessons learned by other Standard Set implementers [23].

There is also a need for flexibility, as ‘ideal’ methods of data collection may not work well in practice. This is particularly relevant for busy public hospital outpatient settings, where data collection may not be feasible within a clinic appointment. A range of data collection approaches (for example, offering both online and paper-based questionnaire completion) may be required to best meet the needs of patients and overcome practical issues. In our study it was evident that paper-based data collection was most popular among our participants. However, this was a very time-consuming approach and unlikely to be manageable in the longer term as patient numbers grow. The level of staffing required to support this approach is reflected in our implementation costs, although there may be economies of scale (after efficient systems for data collection and follow up have been established and patient numbers increase) which would likely reduce the cost per patient over time. It is important to understand the clinical environment well, in order to design data collection procedures that are practical and achievable. Knowledge of staffing levels within outpatient clinics (both clinical and administrative staffing), existing workflows for patient assessment and review, and the patient’s journey through the hospital system (e.g. the timing of clinic appointments, surgery scheduling procedures, and availability of data from hospital databases) is required.

Furthermore, it is important to understand your patient population well. Particular consideration should be given to culturally and linguistically diverse populations who may be unable to complete the ICHOM Standard Set in English. As shown in our study, almost one-third of patients undergoing joint replacement surgery at the RMH were non-English-speaking. While translated versions of some of the individual PROMs instruments are available, the full Standard Set has not been translated into languages other than English. Until translated versions are available, use of a family member or friend as a proxy, or use of interpreter services, may be required.

We also seek to share the key challenges we faced during this implementation study. We used existing resources (with regard to available hardware, software, and IT support) to develop iPad and web-based interfaces for collecting PROMs data. In view of financial constraints, a commercial package for PROMs data collection (e.g. from an ICHOM-recommended technology provider) was not sought. This approach provided us with the flexibility of making changes to the data collection interfaces in response to patient and clinician feedback. However, once our IT support ceased, further development of our web-based PROMs collection portal was not possible and so we returned to paper-based data collection. The volume of missing data (non-returned paper questionnaires as well as initial missing item responses) was problematic, and necessitated a significant time commitment to follow up by telephone and/or mail. This is not only a resource issue, but could have potential implications for data quality. Follow-up of missing data and non-returned questionnaires was most difficult during periods of staff leave. However, the effort invested was worthwhile, as evidenced by the completeness of our baseline data. Missing data can be minimised through electronic data collection (using programming logic that does not allow an individual to proceed to subsequent items if missing responses exist; although there is still the possibility that patients will exit the data collection platform prematurely).

We acknowledge that barriers to Standard Set administration may vary across public and private hospital settings; however, our study was designed to provide an overall perspective of implementation experiences rather than compare the hospital settings. In our experience, clinicians found it challenging to administer the PROMs in the busy pre-admission and outpatient orthopaedic clinic environments, and this likely reflects competing clinical and administrative priorities. There were also significant time restrictions within the outpatient clinic setting and some patients experienced difficulty completing the Standard Set items during their time in clinic. These challenges may be associated with the length of the Standard Set, as it includes a significantly greater number of items than PROMs previously collected at these hospital sites. This is in contrast to an implementation case study involving the Standard Set for Coronary Artery Disease, where the ICHOM dataset was noted to be shorter than the dataset previously collected [24]. We were required to manually extract Standard Set clinical, mortality and readmission data from the hospital databases, as an automated system for extracting these data does not currently exist. It was also challenging to collect some of the required ICHOM clinical variables, for example the 30-day mortality data, as this information is not routinely reported to treating hospitals in Australia. Collection of accurate 30-day all-cause readmission data and ongoing re-operation data is also limited as patients may have presented to other hospitals for treatment of post-operative complications.

We acknowledge that the costs incurred during our implementation and ongoing data collection may differ to those that may be incurred at other hospitals due to a range of factors (for example, depending on available clinical and administrative support, available IT support and patient numbers). In this study, IT support encompassed the development of both iPad-based and web-based data collection platforms and this is reflected in our aggregate costs. However, the growing popularity of online data collection (and mindful of the need, within patient-centred care, to offer alternative approaches for patients who cannot or prefer not to access these methods) supports our costing approach. Our cost estimates can be used to inform implementation decisions by clinicians and policymakers as to whether a similar financial investment would be worthwhile in their own context.

## Conclusions

The ICHOM Standard Set for Hip and Knee Osteoarthritis was launched in mid-2015. However, until now there have been no reports of its implementation or performance in clinical settings. It is only through real-world implementation projects such as ours that we can develop an understanding of how the Standard Sets function in practice, and whether data collection can be feasibly sustained over time. It is likely that feedback from Standard Set users will contribute to the refinement and modification of these measurement sets, and we would recommend shortening the Standard Set to focus on key patient-reported and clinical items. This would undoubtedly facilitate its use in busy clinical settings. Our recommendation would be to remove the treatment progression (types of treatments used previously) and care utilisation (previous use of health professional services) variables as these items are potentially subject to recall bias and classification bias and to remove the all-cause mortality item (particularly as osteoarthritis and its treatments are associated with relatively low mortality) as these data can be difficult to source, depending on the jurisdiction. We also recommend future translation of the Standard Set into languages other than English to ensure that data can be collected from representative patient populations. The sustainability of ongoing Standard Set data collection at our hospital sites will largely depend on the ability to respond to the key learnings from this study; in particular, the availability of dedicated staffing and the capacity to incorporate the required workload within existing clinical roles. Access to appropriate data capture and reporting software, together with adequate IT support, is also needed. Finally, there needs to be a perception of value among clinicians and hospital management who will want to see a tangible benefit from their investment of time and significant financial resources.

## Additional file

Additional file 1: Interview schedules. (DOCX 20 kb).

## Abbreviations

EQ-5D: EuroQoL 5-dimension health-related quality of life instrument
HOOS: Hip Disability and Osteoarthritis Outcome Score
HOOS-PS: Hip Disability and Osteoarthritis Outcome Score - Physical function short form
ICHOM: International Consortium for Health Outcomes Measurement
IT: Information technology
KOOS: Knee Injury and Osteoarthritis Outcome Score
KOOS-PS: Knee Injury and Osteoarthritis Outcome Score - Physical function short form
MAPT: Multi-Attribute Prioritisation Tool
MPH: Melbourne Private Hospital
NRS: Numeric Pain Rating Scale
OA: Osteoarthritis
PROMs: Patient-reported outcome measures
RMH: The Royal Melbourne Hospital
SF-12: Short Form 12 Health Survey
VAS: Visual Analogue Scale
VR-12: Veterans RAND 12 instrument
