# Supplementary material for: The feasibility of implementing the ICHOM Standard Set for Hip and Knee Osteoarthritis: a mixed-methods evaluation in public and private hospital settings
Source: J Patient Rep Outcomes. 2018 Aug 1;2:32. doi: 10.1186/s41687-018-0062-5 (PMC6091617; doi:10.1186/s41687-018-0062-5)
Supplement: Supplementary file 1 — Interview schedules. (DOCX 20 kb). [file 41687_2018_62_MOESM1_ESM.docx]

**Additional file**

**Feasibility and costs of implementing the ICHOM Standard Set for Hip and Knee Osteoarthritis: A mixed-methods evaluation in public and private hospital settings**

Author information removed for blinding purposes

Semi-structured interview schedule (patients)

1. Knowledge regarding use of questionnaires
   1. What is your understanding of why we ask patients to fill in questionnaires before and after joint replacement surgery?
2. Experiences in completing questionnaires
   1. Overall, what was your experience in filling in the questionnaires before and after your surgery?
   2. How did you feel about the time commitment involved?
   3. Was there anything that made it difficult for you to fill in the questionnaires before your surgery?
   4. Was there anything that made it difficult for you to fill in the questionnaires after you had your surgery?
   5. Is there anything that would make it easier for you to fill in the questionnaires?
   6. How would you prefer to receive the questionnaires (e.g. online, paper-based, sent to home, in clinic)?
3. Perceived benefits
   1. Do you see any potential benefits, for you personally, from filling in these questionnaires before and after surgery? (And what might these be?)
   2. Do you see any potential benefits, for healthcare providers (such as the orthopaedic surgeons and physiotherapists), from patients filling in these questionnaires before and after surgery? (And what might these be?)
   3. Do you see any potential benefits, for the hospital, from patients filling in these questionnaires before and after surgery? (And what might these be?)
4. Sustainability
   1. For future patients having joint replacement surgery, do you have any suggestions for how we could improve the experience in regards to these questionnaires?

Semi-structured interview schedule (other stakeholders)

1. Attitudes towards patient reported outcome measures (PROMs)
   1. What were your experiences in administering PROMs prior to this project?
   2. What are your opinions regarding the use of PROMs before and after hip and knee joint replacement surgery?
   3. What do you perceive are the main benefits of using PROMs for:
   - Clinicians?
   - Patients?
2. Implementation Process
   1. What was your understanding of the main aims of the project?
   2. What did you think about the pre-implementation education session/s?
   3. What did you think about the resources provided for the Standard Set implementation?
   4. How could the pre-implementation education and resources be improved?
3. Uptake
   1. Can you comment on your perceived competency administering PROMs?
   2. Can you tell me about anything that makes / has made it easier for the ICHOM Standard Set to be administered in your clinical area during the implementation period?
   3. Can you tell me about any barriers to the ICHOM Standard Set being administered in your clinical area during the implementation period? (e.g. IT resources, time commitment, integration into routine clinical practice, support services during clinics)
4. Sustainability
   1. How sustainable is routine administration of the ICHOM Standard Set in clinical practice at RMH/MPH?
   2. What is needed to facilitate the ongoing use of the ICHOM Standard Set at RMH/MPH?
5. Comparison with previous PROMs collection experiences
   1. Can you describe any differences with the collection of the ICHOM Standard Set during this implementation project, compared with your previous experiences or PROMS use?
   2. What were the positive experiences related to this project?
   3. What areas could be further developed or improved?
